# Interface-exfoliated graphene-based conductive screen-printing inks: low-loading, low-cost, and additive-free

**DOI:** 10.1038/s41598-020-74821-3

**Published:** 2020-10-22

**Authors:** Feiyang Chen, Deepthi Varghese, Sean T. McDermott, Ian George, Lijiang Geng, Douglas H. Adamson

**Affiliations:** 1 Chemistry Department, University of Connecticut, Storrs, 06269 USA; 2 Polymer Program, University of Connecticut, Storrs, 06269 USA; 3 Department of Allied Health Science, University of Connecticut, Storrs, 06269 USA

**Keywords:** Materials for devices, Nanoscale materials, Graphene, Synthesis and processing

## Abstract

Paper diagnostics are of growing interest due to their low cost and easy accessibility. Conductive inks, necessary for manufacturing the next generation diagnostic devices, currently face challenges such as high cost, high sintering temperatures, or harsh conditions required to remove stabilizers. Here we report an effective, inexpensive, and environmentally friendly approach to graphene ink that is suitable for screen printing onto paper substrates. The ink formulation contains only pristine graphite, water, and non-toxic alkanes formed by an interfacial trapping method in which graphite spontaneously exfoliates to graphene. The result is a viscous graphene stabilized water-in-oil emulsion-based ink. This ink does not require sintering, but drying at 90 °C or brief microwaving can improve the conductivity. The production requires only 40 s of shaking to form the emulsion. The sheet resistance of the ink is approximately 600 Ω/sq at a thickness of less than 6 µm, and the ink can be stabilized by as little as 1 wt% graphite.

## Introduction

Paper diagnostics were first introduced in 2007 as a potential solution to high cost and often inaccessible medical diagnostics^1^. They have drawn interest because paper is inexpensive, absorbs fluids, can be easily modified, disposed of, and scaled up^2^. Important applications include diagnosing often neglected tropical diseases, home-based cancer and liver diagnostics, food and water testing, and environmental quality monitoring in developing countries^2,3^. As the low cost of these devices is critical to their utility, future paper diagnostics will require inexpensive, non-toxic, and easily processed conductive inks. Currently conductive inks find applications in flexible energy storage materials^4–6^, sensors^7,8^, antennas^9^, and electronics^10,11^. Metal particle-based conductive inks dominate the market, and although these inks provide high electrical conductivity, they are expensive, often unstable and toxic, and require high sintering temperatures^12–18^. To address these challenges there has been a great deal of effort in developing carbon-based, and more specifically graphene-based, inks^19–21^. However, difficulties in exfoliating graphite and suspending graphene have made these inks expensive, as evidenced by the cost of commercial graphene-based ink marketed for screen printing exceeding $500 per 10 ml. (Graphene ink in water, flexo/gravure/screen printable, Sigma Aldrich).

There are two basic approaches to the use of graphene for conductive inks. The first is to reduce graphene oxide (GO) after printing, either by thermal or chemical treatment. This approach requires harsh oxidation of the graphite, but provides water suspendable material^5,7,8^. The second approach uses liquid-phase exfoliation with added stabilizer to keep the graphene suspened^4,22,23^. The GO reduction method addresses the challenge of suspending graphene by adding hydrophilic functional groups to the graphene sheets. This introduces defects but sacrifices the electrical conductivity, which is partially restored by the reduction step^23–25^. In addition, the method often used for making GO involves harsh chemical treatment in concentrated sulfuric acid^7,26^, and the substrates suitable for GO inks are limited due to the GO reduction process that can require post-print temperatures from 200 to 450 °C^27,28^.

The alternative to the reduction of GO is liquid-phase exfoliation. In this approach graphite is tip sonicated, often at high power for several days in high boiling temperature solvents such as N-methyl-2-pyrrolidone (NMP), dimethylformamide (DMF), or surfactants^4,9,10,29,30^. This is then followed by further stabilization of the graphene by the addition of chemical binders such as polymethyl methacrylate (PMMA), polyvinyl alcohol (PVA), polyvinyl pyrrolidone (PVP), or ethyl cellulose (EC)^10,23,31,32^. However, this results in the need for annealing at temperature from 250 to 400 °C to remove the non-conductive binders after printing^23,33–35^.

An ideal ink would consist of unoxidized, exfoliated graphene sheets without hard-to-remove suspension agents. Here we report an approach to create such an ink using interfacial trapping. The final inks contain only water, graphene, and alkanes and are prepared by simple shaking for 40 s using a bubble tea shaker. The inks are thus inexpensive and promising for large scale production. Shaking the mixture of water, graphite, and alkane forms viscous water-in-oil emulsions stabilized by exfoliated graphene sheets at the water/oil interface. No toxic chemicals or additional stabilizers are needed. The solvents used can be removed either by drying at 90 °C or by microwaving for 40 s. The inks reported here have a graphite loading of 0.1 g/10 ml, but higher and lower concentrations are also stable.

Since the exfoliation of the graphite is driven by lowering the interfacial energy of the oil/water interface, once sufficient graphene has been produced to cover the interface, exfoliation does not continue. This results in both graphite and graphene being present at the interface. The presence of graphene, and not just graphite, has been shown by TEM images and Raman spectroscopy^36^. Other studies have shown that graphene alone can stabilize emulsions, such as the work of Large et al., where graphite was exfoliated by sonication and used to stabilize water-in-oil emulsions^37^, and the work of Ogilvie et al. who pre-exfoliated with sonication followed by size selection to produce stabilized emulsions with only few layer graphene^38^. These emulsions were shown to require low graphene loading. In addition, a recently published study demonstrates that graphene stabilizes water-in-oil emulsions due to the amphiphilic nature of pristine graphene, and that based on DFT calculations dispersive interactions between stacked plates reduces the amphiphilic nature of single flakes, meaning that graphite flakes by themselves cannot stabilize water/oil emulsions^39^. Other research also supported this idea by successfully forming water-in-hexane emulsions using liquid-phase exfoliated graphene^38^.

A recent review of graphene based conductive inks compared them in terms of their reported sheet resistance^10^. Values ranged from ~ 2 Ω/sq up to ~ 2 × 10^5^ Ω/sq with a median value of 260 Ω/sq. The variation in these values is at least partially a result of a range of approaches, thicknesses, and conditions used for measuring sheet resistance. All of these inks also contained a stabilizer, often cellulose, that required removal by high temperature annealing, often higher than the autoignition temperature of paper. Our measured sheet resistance of ~ 600 Ω/sq is comfortably in the range of other reported inks, despite the lack of a high energy exfoliation method as was required for all the other reviewed inks. Additionally, the lack of an added stabilizer eliminates the need for high temperature annealing, making our inks an attractive option for paper based electronics.

## Results and discussion

**Figure 1:**
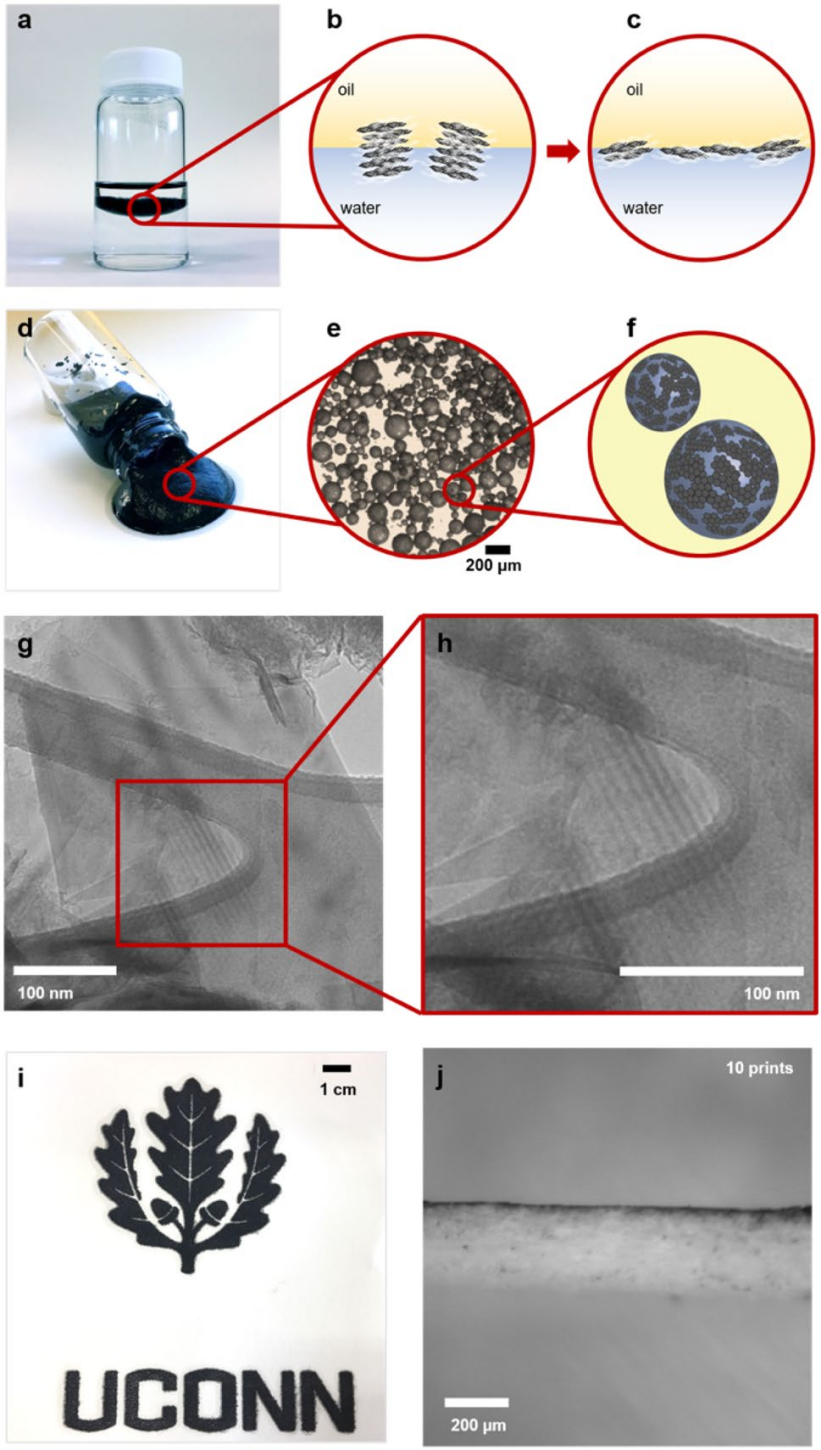
(**a**) Vial containing hexadecane (top), water (bottom), and graphite. Although denser than water, the graphitic material is pinned at the oil/water interface; (**b**) illustration of graphite initially at the oil/water interface; (**c**) overlapping graphene sheets at the solvent interface after the spontaneous spreading (exfoliation) of the graphite; (**d**) conductive ink consisting of graphene stabilized water-in-oil emulsion; (**e**) optical microscopy image of the ink showing graphene stabilized water droplets; (**f**) illustration of water-in-oil emulsion with graphene stabilizing the interface; (**g**) TEM image of graphene prepared by interfacial trapping showing overlapped exfoliated sheets; (**h**) magnified image showing the moiré pattern on graphene formed by re-stacked exfoliated graphene sheets; (**i**) UCONN (University of Connecticut) logo printed on paper using graphene emulsion ink; (**j**) A cross-section of graphene emulsion ink printed on paper.

Exfoliating graphite by interfacial trapping relies on graphite not being soluble in either water or oil^36^. The exfoliation is spontaneous and driven by lowering the interfacial energy of oil and water as graphene spreads at the interface^36,40^. Shown in Fig. 1a is a vial containing hexadecane (C16) and water with graphite trapped at the oil/water interface. When a graphite flake encounters the oil/water interface (Fig. 1b) it exfoliates, with the individual graphene sheets spreading to cover the interface as depicted in Fig. 1c^36,40–43^.

Emulsifying the system by shaking or stirring produces new oil/water interfaces. This in turn drives further exfoliation of the graphite as it covers the new interfaces, leading to a water-in-oil emulsion with graphene sheets stabilizing the interface. Figure 1d shows a viscous graphene stabilized water-in-oil emulsion made by hand shaking the scintillation vial in Fig. 1a. This emulsion has suitable viscosity for screen printing without the need for any chemical binders. Furthermore, the ink only contains 1 wt% of graphene, while typical electrically conductive screen printing inks require graphene loading as high as 78 wt% in addition to chemical binders^44^. Diluting the ink with additional continuous phase allowed the spheres to be observed under an optical microscope as shown in Fig. 1e. Figure 1f illustrates the general morphology of the emulsion with graphene sheets stabilizing the water droplet surface.

Diluting the ink with excess dispersed phase broke the emulsion and resulted in a film of overlapping graphene sheets at the water surface. Transmission electron microscopy (TEM) was used to image this film. Figure 1g shows 2–3 layers of overlapping graphene sheets formed by exfoliated sheets that partially restacked. Restacking, rather than having not been exfoliated, is indicated by the moiré pattern shown in Fig. 1h. This moiré pattern forms when overlapping graphene sheets are stacked at a twisted angle^45–48^. This would not be expected in the original graphite.

Printing with this graphene ink does not involve any modification to the screen-printing stencil. This viscous graphene ink can print into complicated patterns with good resolution as shown in Fig. 1i. These lines printed on paper are electrically conductive and only partially adsorb below the surface of the paper. Figure 1j is a cross-section image of the ink printed on paper. The ink only penetrated the substrate by a few microns even after 10 prints. In the following study, we investigate the kinetics, resolution, viscosity, and electrical conductivity of these remarkable, inexpensive, and easily scalable exfoliated graphene-based inks based on a fundamentally new approach to graphene exfoliation and solution stabilization.

**Figure 2:**
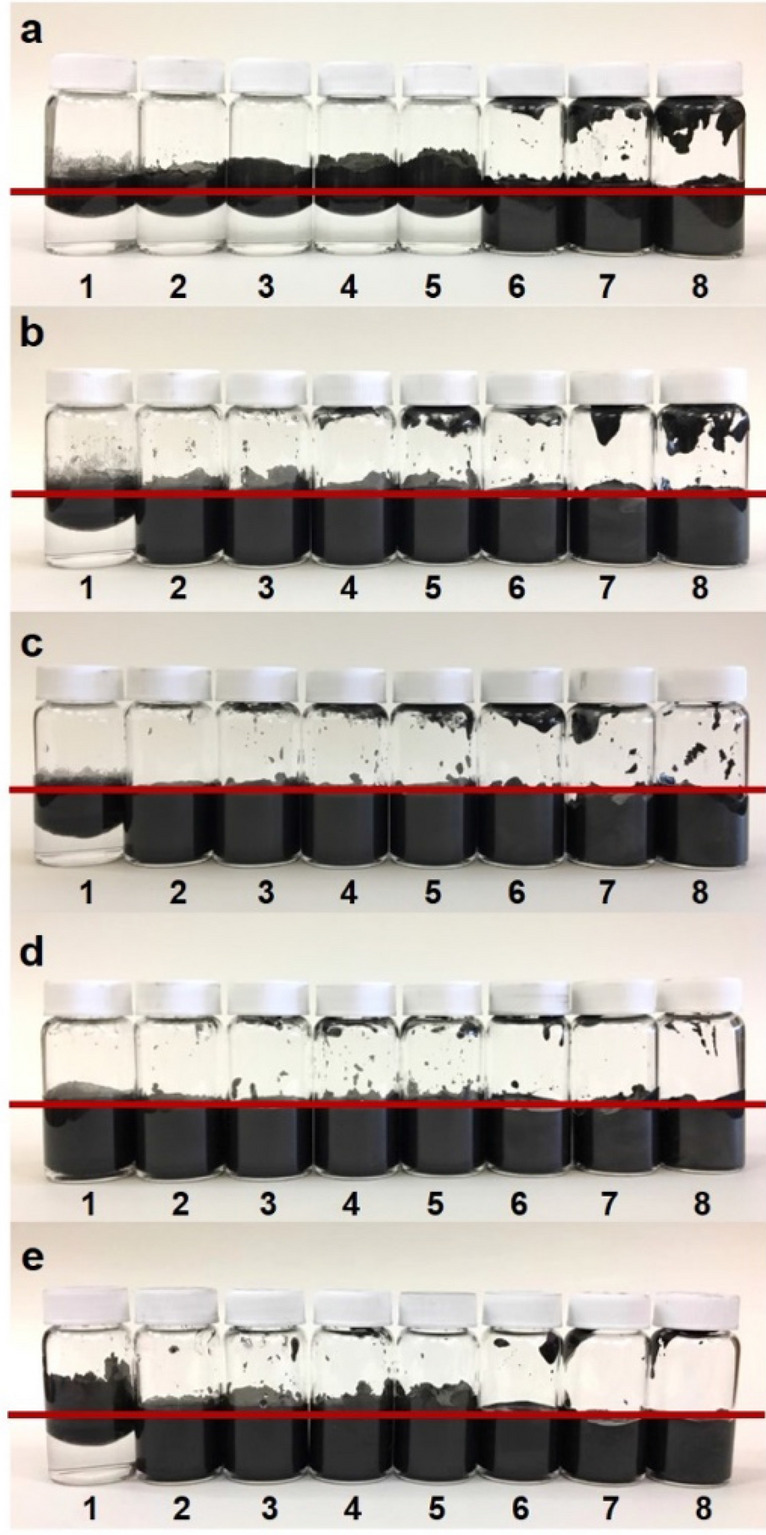
From left to right vails contain increasing concentration of graphite. From vial 1–8 (in g/ml): 0.001, 0.002, 0.003, 0.004, 0.005, 0.010, 0.020, 0.030. The solvent is a 7:3 volume ratio of water and hexadecane (red line indicates the liquid/air interface); (**a**) emulsions immediately after shaking for 40 s; (**b**) same samples as in a after sitting for 24 h followed by a second 40 s shaking; (**c**) emulsions from (**b**) after standing for an additional four days, then shaken a third time for 40 s; (**d**) same samples as in (**c**) after standing an additional 7 days (12 days since initial shaking) followed by a fourth shaking for 40 s; (**e**) emulsions immediately after shaking for 160 s.

The rate of emulsion formation in the graphene inks is different than with typical emulsions. Exfoliation of the graphite is not instantaneous, meaning that in some cases there initially is insufficient graphene to stabilize a full emulsion. To study this effect, different graphite loadings were investigated. As shown in Fig. 2a, eight vials with increasing amounts of graphite from left to right were shaken continuously for 40 s using a bubble tea shaker. Any graphene observed above the red line was a result of graphene climbing at the interface of the hydrophilic glass and hydrophobic solvent, and is not considered when analyzing the emulsion volume^36^.

As seen in Fig. 2a, only a partial emulsion was formed in vials 1 to 5 after the first shaking. This is because there was insufficient graphene surface area to stabilize a full water-in-oil emulsion, but at higher initial graphite loadings there was enough surface area to stabilize full emulsions, as seen in vials 6 to 8. The vials were then allowed to sit for 24 h, and shaken again for 40 s (Fig. 2b). In this case, all but vial 1, with the lowest graphite loading, formed full emulsions. However, the emulsion volume of vial 1 did exhibit an increase. After 5 days, vial 1’s emulsion volume continued to increase after all vials were shaken again for 40 s (Fig. 2c).

At day twelve, all vials were shaken again for 40 s, and vial 1 formed a full emulsion (Fig. 2d). This suggests that there was some amount of unexfoliated graphite in the vials after the initial emulsification, and that the graphite continued to exfoliate over time. TEM images (Figure S1) confirmed that in addition to the graphene sheets, there was some unexfoliated graphite which could exfoliate further and cover new interface.

It is important to note, however, that the same emulsion volume could not be achieved without the time gaps between shaking. In Fig. 2e, all eight vials were continuously shaken for 160 s, but the graphene exfoliation extent was less than observed in Fig. 2d, even though both sets of vials underwent the same amount of shaking. The extent of graphite exfoliation in Fig. 2e was similar to that observed in Fig. 2b, where each vial had received only half the amount of shaking, but with a 24 h gap between shakes. The results of this kinetic study suggest that the initial coverage of graphene at the water/oil interface is not 100% in a freshly formed emulsion. Rather, it is some minimum value required for emulsion stabilization. Since the graphene exfoliation is a spontaneous process, the unexfoliated graphite exfoliates further to cover a higher percentage of the available interface until reaching a maximum. Then, when shaken again the next day, more emulsion can be immediately formed by the graphene in excess of the minimum required for stability.

**Figure 3:**
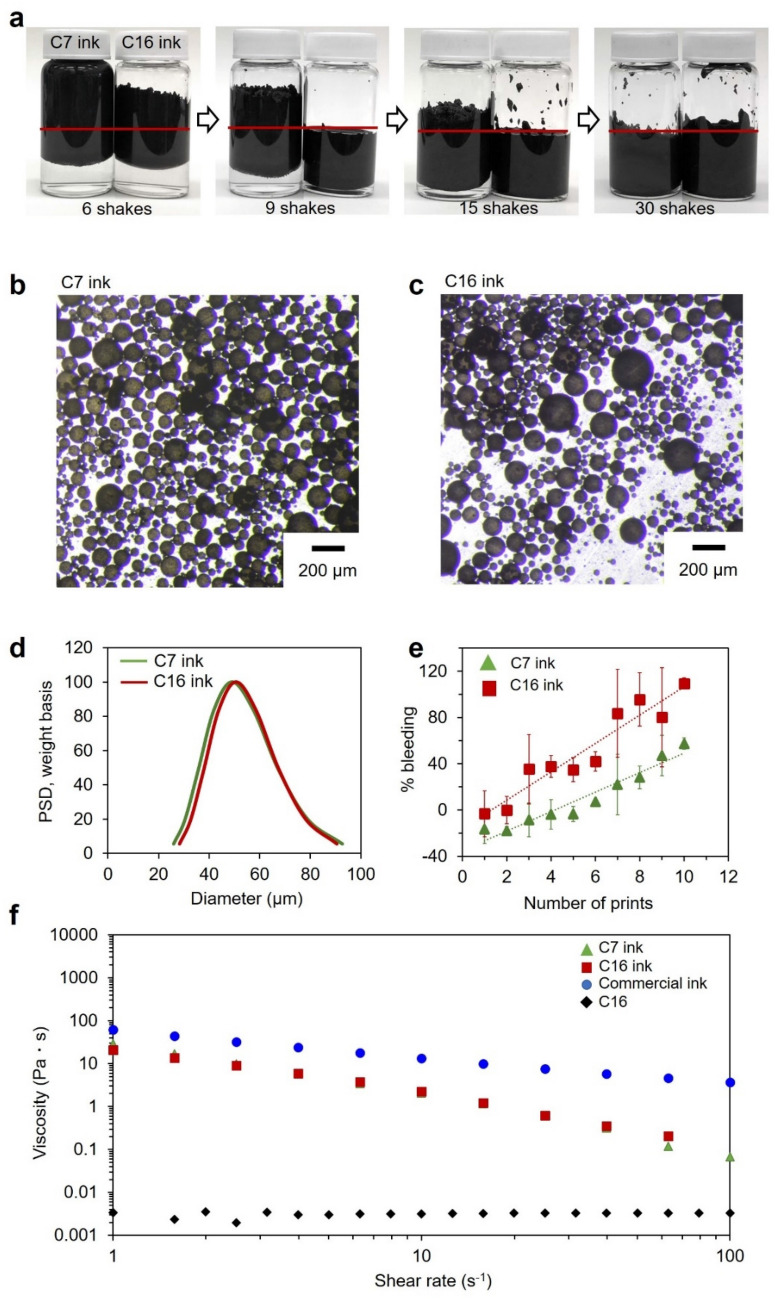
(**a**) Graphene stabilized emulsion forms faster with C16 as compared to C7. (red line indicates the liquid/air interface, C7 represents heptane, C16 represents hexadecane); (**b**) optical microscopy image of C7 ink; (**c**) optical microscopy image of C16 ink showing water droplets to be approximately the same size as seen with C7 in (**b**); (**d**) acoustic spectroscopy results showing similar size and size distribution of C7 and C16 inks; (**e**) plot comparing the line resolution with number of prints for C7 and C16 inks. The error bars are the standard error calculated from a minimum of three measurements for each data point: (**f**) viscosity of C7 and C16 ink at different shear rates, with a comparison to a commercial screen-printing ink and pure C16.

In addition to the initial loading of graphite, the type of alkane had a significant effect on the rate of emulsion formation. Figure 3a shows the results of using heptane (C7) and C16 as the oil phase to form the emulsion. The C7 ink showed a slower emulsion formation rate than the C16 ink. There are several possible explanations for this. Since the graphene exfoliation is driven by lowering the interfacial energy between oil and water, the difference in interfacial energy may lead to a difference in the equilibrium between exfoliated and stacked graphene. The interfacial energy between C16 and water is 55.2 mN/m, which is higher than C7 and water, 51.9 mN/m^49^. So, graphite exfoliation in the C16 ink is more energetically favorable compared to C7, driving the equilibrium towards exfoliated graphene in the C16 ink system.

Another possible explanation involves the sharpness of the oil/water interface. Moiré patterns shown in Figure S2 indicate that with both the C7 and C16 oil phase, the graphite exfoliates to graphene sheets that then overlap with other sheets. Compared to the C7/water interface, the C16/water interface is shaper, with the solubility of water in C16 being 0.00231 mol/L and C7 being 0.00346 mol/L^50^. Since the restacking of graphene requires a sheet to move towards either the oil or water phase and away from its minimum energy location, the steeper rise in free energy associated with a sharper interface should inhibit restacking^26^.

Adsorbed alkane may also inhibit restacking. For alkane absorption on graphene sheets, computational studies have shown that with increasing alkane number there is an increase in bonding energy between the alkane and graphene^51^, meaning that graphene is expected to absorb more strongly C16 than C7. Likewise, computational studies have suggested that as the alkane number increases there is an increase in the desorbing energy between the alkane and graphene^52^. However, despite the difference in emulsion formation rate between the C7 and C16 inks, both had a similar emulsion droplet size, around 50 μm, and a similar droplet size distribution, as observed by optical microscopy (Fig. 3b,c), and confirmed by acoustic spectrometry (Fig. 3d).

In addition to affecting the rate of emulsion formation, the alkane also affected the quality of the printing. Percent bleeding of each line is the ratio of the width difference between the desired and actual printed line to the width of the desired line. Results are shown in Fig. 3e. Bleeding was observed to be near zero percent for one or two prints, increasing as the paper became saturated. While it appeared in Fig. 3e that C7 ink had negative bleeding at small print numbers, this was due to the ink clogging the screen. A significant amount of C7 ink stayed on the screen rather than passing through. This was attributed to the tendency of emulsion spheres in the C7 ink to form aggregates.

This sphere agglomeration in the C7 ink was observed visually. The top surface of the C7 ink emulsion appeared to be rough and uneven in contrast to the very smooth surface of the C16 ink emulsion (Figure S3). Since C7/water has a more diffuse interface than C16/water, adjacent spheres in contact with each other may be more intimately associated, increasing the energy required for separating spheres. When the ink passes through the screen-printing stencil, spheres must separate from each other and new water/oil interfaces have to be formed. For C7 inks, this results in clogging of the screen and an apparent negative percent bleeding. Thus, for a continuous screen-printing application, an ink like C7 ink would not be ideal.

In a screen printing process, the printing speed can vary from less than 1–100 m/min^53^, making the viscosity of the ink a critical parameter. The viscosities of the C7 and C16 inks were measured at shear rates ranging from 1 to 100 s^−1^ by parallel plate rheometry, and the results are compared to a commercial screen-printing ink as shown in Fig. 3f. As a control, the C16 alkane was also analyzed. The graphene inks and the commercial ink showed similar viscosities and shear thinning properties. In contrast to the simple, three-ingredient graphene inks introduced here, commercial inks have complicated formulations, comprising different solvents, binders, pigments, dyes, resins, lubricants, solubilizers, and surfactants in order to achieve the necessary viscosities for screen printing. Even at shear rates higher than 10 s^−1^, the graphene inks have viscosities suitable for screen printing, higher than 0.500 Pa s^54^. Previously reported graphene inks with similar viscosities require 30 times more graphene, > 0.3 g/ml graphene as compared to 0.01 g/ml, to achieve the viscosities reported here^33,44^. A recent study of an emulsion stabilized by only graphene reported a viscosity 100 times less than our inks^7^. This difference is likely due to the presence of some unexfoliated graphite in our system.

The electrical conductivity of conductive inks is typically characterized by measuring the sheet resistance of a printed line using a four-point probe, often combined with the thickness of the line measured by atomic force microscopy (AFM) or profilometry^9,33,42^. However, when the substrate is a porous material like paper, thickness measurements are more challenging, as the conductive particles can penetrate the substrate, as shown in Fig. 1i, or coat the fibers, as shown in Figure S4. In our case the penetration depth of the ink did not appear to increase with additional overlapping prints, but the resistance of the line decreased. Figure S5 shows that the thickness of the printed line appeared to be nearly identical for a line printed once versus ten times. This makes direct conductivity comparisons with previous studies somewhat challenging.

To address the challenge of surface penetration for our conductivity measurements, the C16 ink was drop cast on a glass slide. The sheet resistance of the ink was found to be approximately 600 Ω/sq. The conductivity was calculated using a thickness value based on the mass of graphene, size of the ink dot on the glass slide, and the density of graphite (For details see Figure S6). Conductivity of the C16 ink was found to be as high as 280 S/m. The printed ink was also flexible, with little increase in resistance after repeated bending. Wrapping a printed line around a 7.62 cm diameter tube only increased the resistance by 35% after the first 15 cycles, with little to no change after an additional 100 cycles. The results are shown in Figure S7.

**Figure 4:**
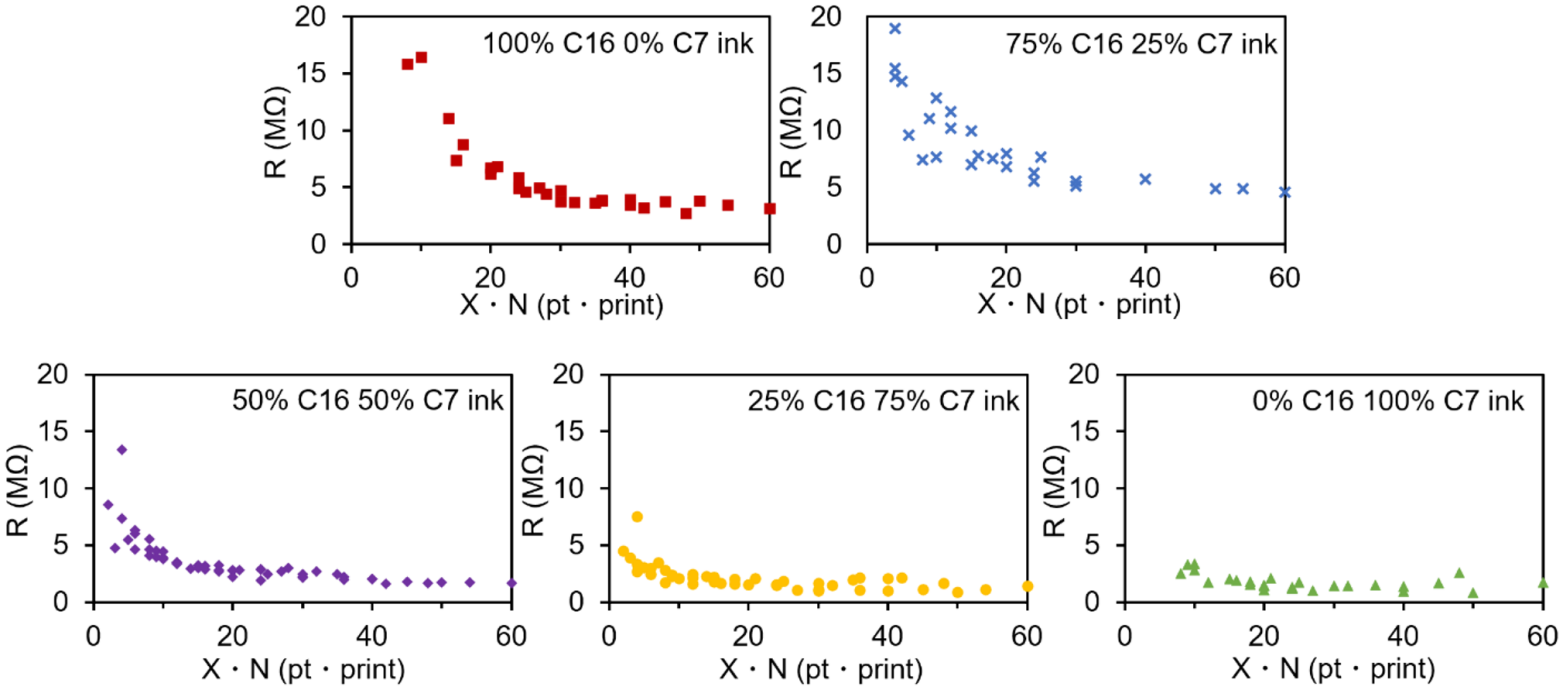
The resistance of printed lines on paper prepared with inks of varying proportions of C7 and C16. Resistance is plotted as a function of the product of line width (X) and the number of prints (N).

An important parameter for conductive inks is the change in resistance with increasing line width (X) or an increasing number of prints (N)^20,44^. A decrease in resistance would be expected with increases in both X and N. While results in Fig. 4 show this to be the case, the composition of the oil phase proved to be surprisingly important. When the oil phase contained more C16, there was an overall increase in the resistance of the ink line. This appeared to be due to the low volatility of C16, as the lines were only dried overnight at 90 °C, leaving residual C16 that separated the sheets from one another. This was especially true for small values of X•N, where the resistance of the 100% C16 ink lines was too great to be measured.

That residual C16 was the cause of the high observed resistance was shown by further drying the printed lines. When the lines were dried for a week at 90 °C, the C16 ink resistance was decreased to roughly a third of the original resistance. In addition, the resistance of C16 ink lines was also reduced by roughly 70% after microwaving the printed lines for four cycles of ten second duration, with the results shown in Figure S9.

While the lack of conductivity in the C16 inks was a result of low vapor pressure, the reason for the lack of conductivity of the C7 inks at small values of X•N is not as obvious. Visual observation of lines made with C7 suggested that at low print numbers C7 inks were less uniform than C16 inks. Further, as mentioned previously, vials of inks made with C16 appeared to have smooth surfaces while inks made with C7 had uneven or rough surfaces. The presence of aggregates in the C7 ink may cause non-uniform application.

**Figure 5:**
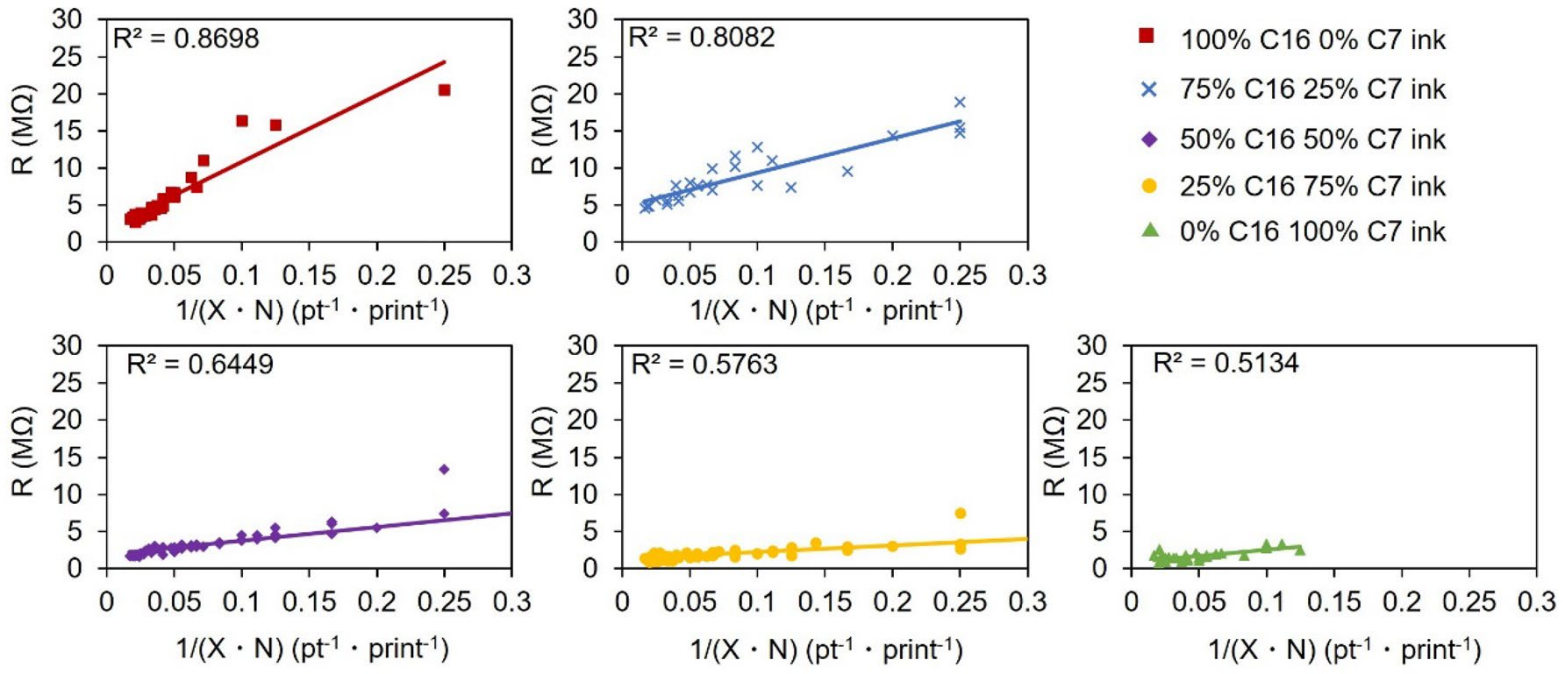
Comparison of the linearity of resistance verses 1/(X•N) plots of inks made with different C7/C16 ratios.

This non-uniform application was quantified by replotting the data derived from nearly 400 individual measurements in Fig. 4 to the linear plots shown in Fig. 5. Based on Eq. (1), where A indicates the effect of additional graphene on the resistance of the printed line. Resistance was plotted as a function of the inverse of X•N, and the linear fit of the lines was compared. If the ink application was uniform, each print should transfer the same mass of graphene to the substrate. Deviations in the amount of graphene transferred per print, or in the uniformity of the graphene application, should result in deviations from linearity and thus smaller values of the coefficient of determination (R^2^). It was observed that with increasing C7 percentage in the ink, there was a steady decrease in the value of R^2^, providing evidence that the C7 inks provide less uniform graphene application during screen printing.

1 $$R = \frac{1}{X \cdot N}A$$

**Figure 6:**
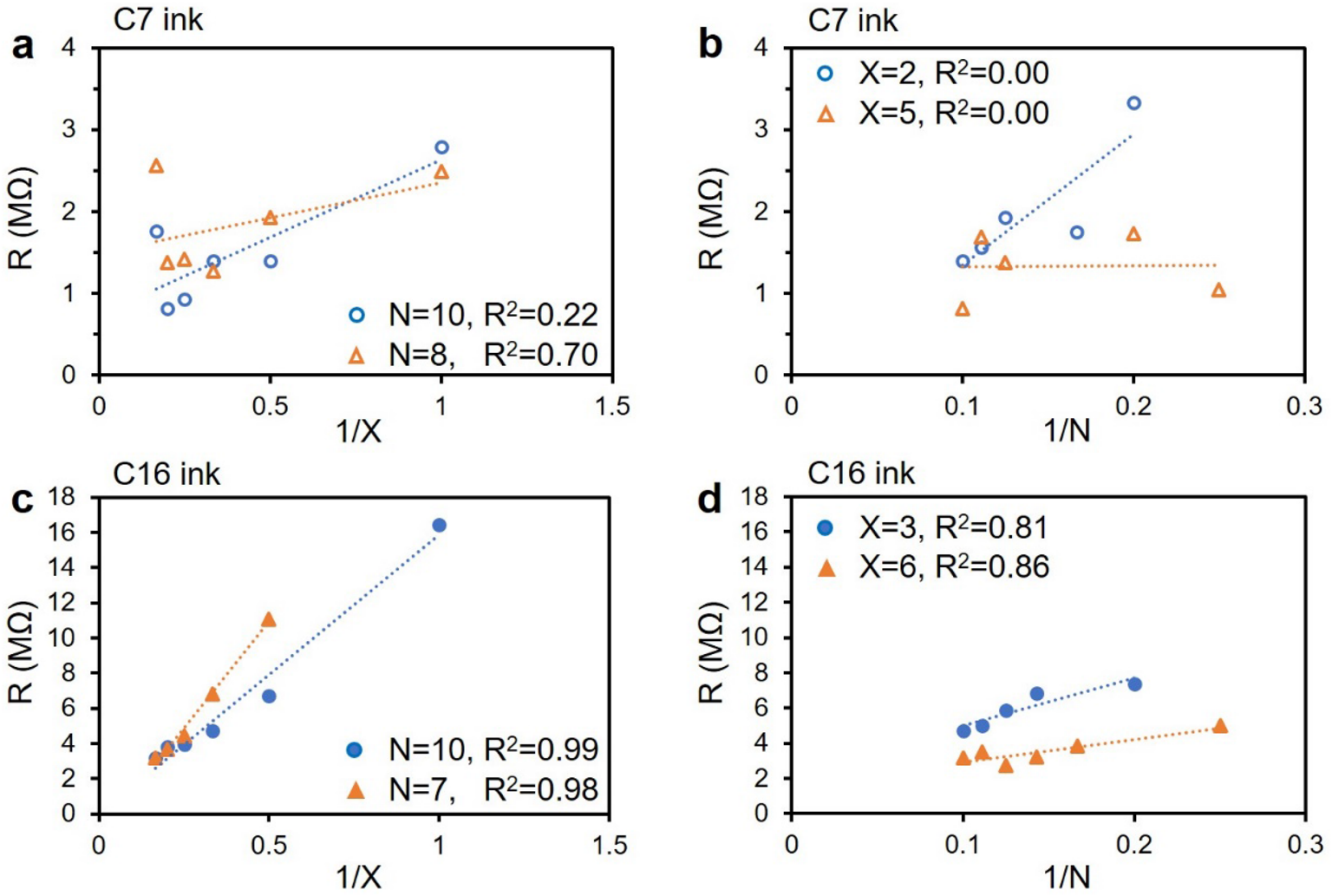
(**a**) Resistance of the C7 ink vs. 1/X (pt^−1^) when N (print^−1^) is constant; (**b**) resistance of the C7 ink vs. 1/N when X is constant; (**c**) resistance of the C16 ink vs. 1/X when N is constant; (**d**) resistance of the C16 ink vs. 1/N when X is constant.

The cause for the less than perfect R^2^ values in the C16 rich inks and the very poor R^2^ values in the C7 rich inks was investigated by comparing 1/X and 1/N separately, as shown in Fig. 6. In the case of the C7 ink, there is poor correlation of resistance with line width, and nearly no correlation of resistance with the number of prints. This suggests that the non-uniformity of C7 inks is more a result of the ink not passing through the screen than the ink not spreading when in contact with the paper. In contrast, the C16 line resistance correlates very well with the width of the line, and somewhat less with the number of prints. Any non-uniformity in the lines printed with C16 ink appears to be a result of slight variations in the amount of graphene applied during each print.

## Conclusions

Here we demonstrated a graphene-based screen-printing ink prepared by interfacial trapping of untreated graphite using non-toxic chemicals. The ink can be prepared in as little as 40 s by simple shaking of a graphite/water/alkane mixture. In contrast to other approaches, no high energy step such as tip sonication or high shear mixing was involved, only a bubble tea shaker set at 320 rpm. Graphite loading in the ink was as low as 0.01 g/ml with no post-printing procedures requiring harsh chemical treatment or high temperatures as no high boiling point chemical binders or stabilizers were involved in the formulation. The solvents, water and an alkane, can be effectively removed at 90 °C or with 40 s of microwaving. The ink demonstrated excellent electrical conductivity of approximately 300 S/m, with good bending tolerance.

The rate of ink formation was shown to be affected by the alkanes used, with the C16 based ink forming faster than the C7 based ink. A statistical approach for analyzing the correlation of the ink’s resistance with the number of prints and the width of the printed line was introduced, providing information about how the uniformity of the ink related to its mechanism of formation. The rheological properties of the ink indicated a wide range of suitable printing speeds, similar to typical commercially available screen-printing inks. This graphene stabilized emulsion ink demonstrates significant potential for enabling the next generation of paper based diagnostics to improve the medical and environmental diagnostics of underserved populations.

## Methods

### Ink formation

heptane (C7) and hexadecane (C16) ink used for screen printing are made by using 0.1 g graphite (Asbury Carbons, grade Nano 24), 3 ml of heptane (n-Heptane, 99%, Optima, Fisher Chemical) or hexadecane (n-Hexadecane, 99%, pure, Acros Organics), and 7 ml of DI H_2_O followed by a forty-second bubble tea shaker shaking (Happybuy Milk Tea shaker, 320 rpm). The bubble tea shaker mimicked the process of hand shaking but allowed a more reproducible study of the effect of shaking time. An image of the bubble tea shaker can be found in Figure S12. For making larger batch of ink sample, ratio of graphite, water and alkane were scaled accordingly, while the bubble tea shaker shaking time remained at 40 s.

### Optical image of the emulsion ink

Samples for optical image of C7 and C16 ink are made by diluting the ink using the corresponding continuous phase to separate spheres, which means C7 ink was diluted using C7 and C16 ink was diluted with C16. Images were taken using Leica M125.

### Ink printing

The stencil used for screen printing are made using Speedball Diazo Ultimate Screen Printing Kit followed by the instruction. Paper used for screen printing is Heavyweight Drawing from Strathmore. When preparing samples for the electrical conductivity study, each ink was printed on paper from 1 to 10 times using a stencil with designed lines widths from 1 to 6 pt. See Figure S8 for an example print. Ink lines were then dried in a 90 °C oven overnight for conductivity measurements.

### Viscosity measurements

C7 and C16 ink used for viscosity measurements are used as it is once a full emulsion was obtained. Commercial screen-printing ink is a fabric screen printing ink purchased from Speedball. Viscosity of the inks are measured using the rheometer (TA instrument, AR G2 5B2730 rheometer) with a sandpaper (Norton T414 Blue-Bak Abrasive Sheet, Paper Backing, Silicon Carbide, Waterproof) modified parallel plate (40 mm parallel plate, Peltier plate steel). A steady shear test from 0.1 to 200 s^−1^ was performed, and the testing temperature is controlled at 30 °C.

### Acoustic spectrometer measurements

C16 and C7 ink sample were made using the bubble tea shaker to obtain a full emulsion and poured into an DT-1202 Acoustic and Electroacoustic spectrometer sample cell (Dispersion Technology, Inc.). The emulsion sphere size and its distribution were measured, and the attenuation spectra is analyzed using Dispersion Technology software.

### Electrical conductivity measurements

Conductivity of the printed ink line was measured using a two-point probe multimeter (Fluke 25 multimeter), and each measurement was repeated at least three times and an average was taken. Resistance value was reported as a function of the product of the line width and the number of prints. Samples for the ink conductivity measurement were prepared by drop casting it onto a glass slide, and dried at 90 °C in an oven for a week. Sheet resistance of the ink was measured using a four-point probe (Signatone S-302), and the thickness of the ink dot was calculated using the volume of the ink drop casted, loading of the graphite, area of the droplet, and graphene density. Conductivity of the ink is calculated based on the sheet resistance the thickness of the dry ink droplet.

### Transmission electron microscope of exfoliated grapheme

Exfoliated graphene sheet sample was obtained by dropping the ink into a large excess of the water. Emulsion droplets break and forms a graphene skin since the oil phase is no longer the continuous phase. A TEM grid (Ted Pella, “holey” performed carbon film) was used to lift the graphene skin. The graphene sample was characterized using TEM (FEI Lab6 20–120 kV transmission electron microscope) at 80 kV.

## Supplementary information

Supplementary file 1

## Data Availability

The datasets generated during the current study are available from the corresponding author on reasonable request.
