# Supplementary material for: Interface-exfoliated graphene-based conductive screen-printing inks: low-loading, low-cost, and additive-free
Source: Sci Rep. 2020 Oct 22;10:18047. doi: 10.1038/s41598-020-74821-3 (PMC7583245; doi:10.1038/s41598-020-74821-3)
Supplement: Supplementary file 1 [file 41598_2020_74821_MOESM1_ESM.docx]

Supporting information for:

**Interface-exfoliated Graphene-based Conductive Screen-printing Inks: Low-loading, Low-cost, and Additive-free**

Feiyang Chen^1^, Deepthi Varghese^1^, Sean T. McDermott^2^, Ian George^3^, Lijiang Geng^3^, and Douglas H. Adamson^1,2,*^

^1^Chemistry Department, University of Connecticut, Storrs, 06269, USA

^2^Polymer Program, University of Connecticut, Storrs, 06269, USA

^3^Department of Allied Health Science, University of Connecticut, Storrs, 06269, USA

^4^Department of Statistics, University of Connecticut, Storrs, 06269, USA

^*^douglas.adamson@uconn.edu


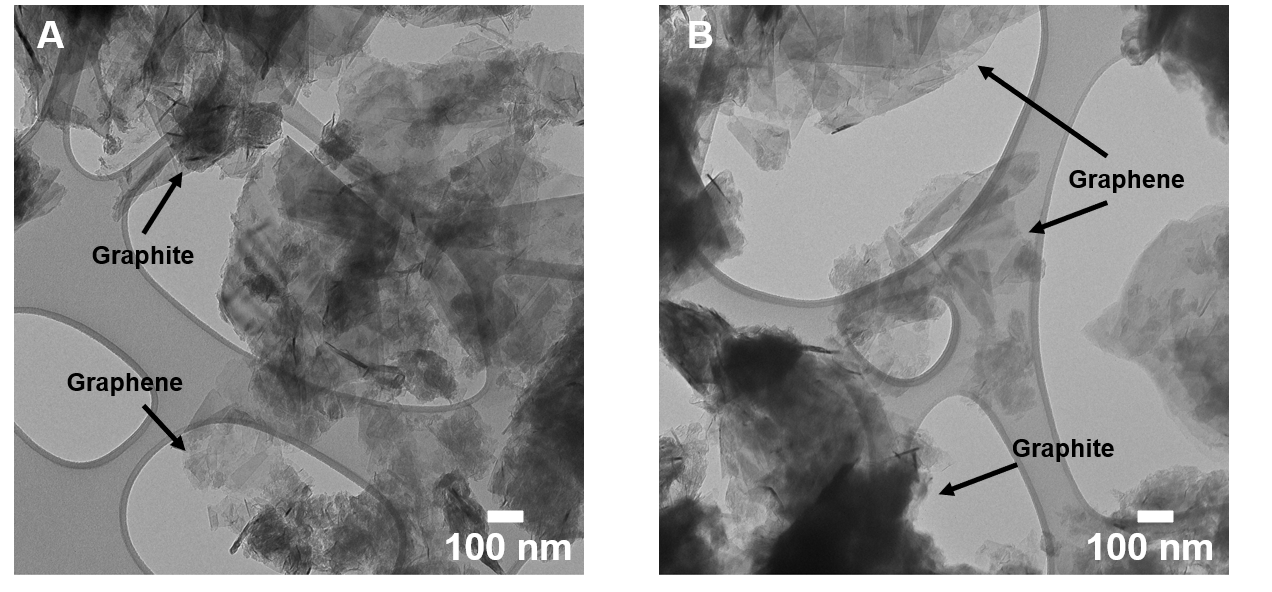


Figure 1. TEM of some few-layer graphene in C7 (A) and C16 ink (B)


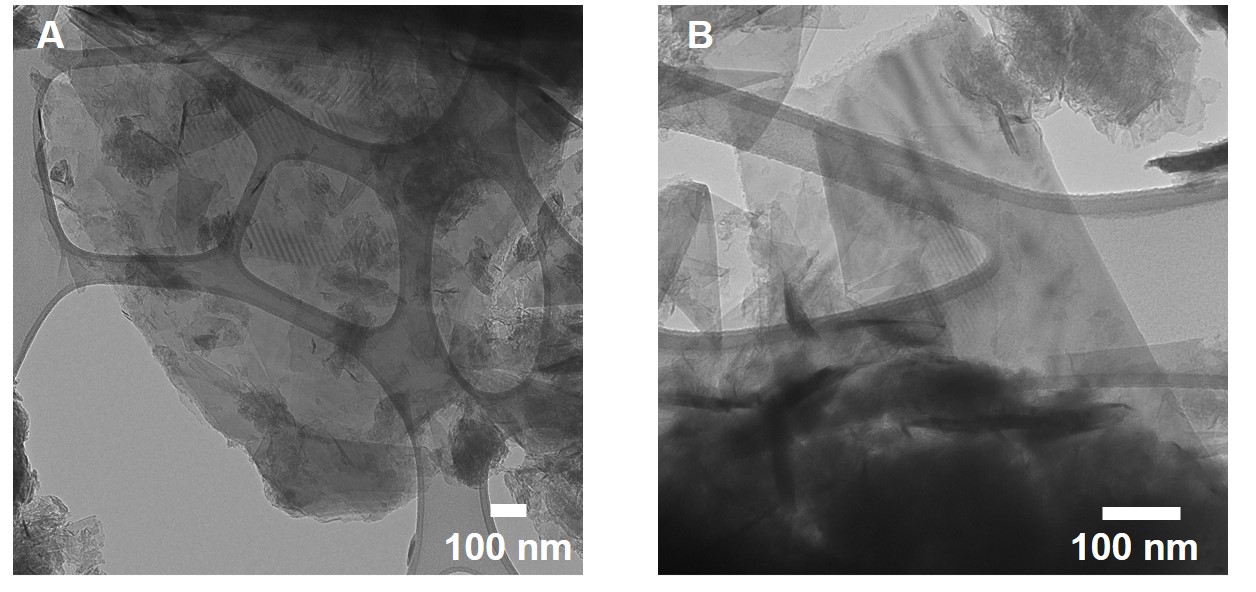


Figure 2. Moiré pattern of graphene in C7 (A) and C16 ink (B) by TEM


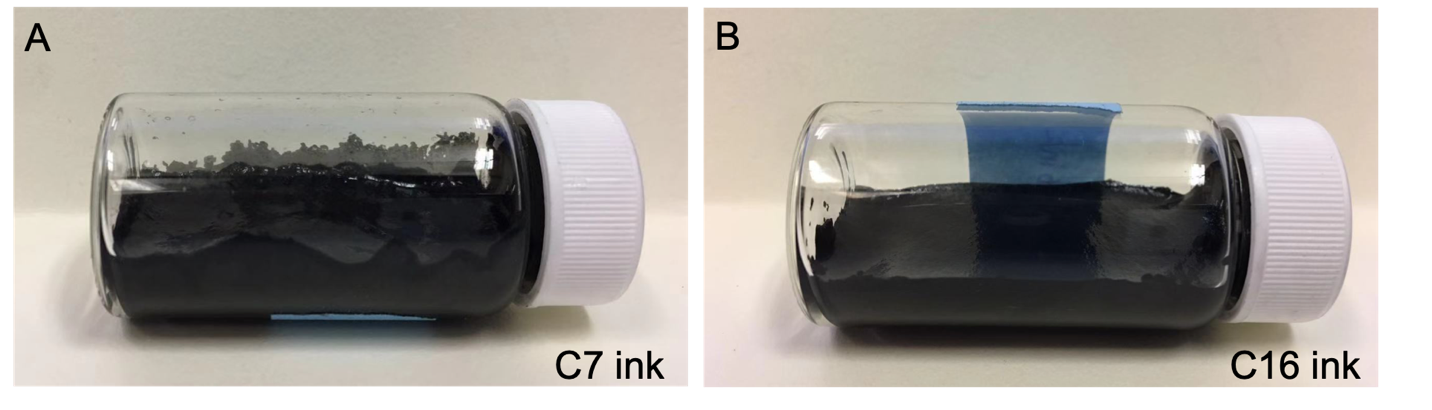


Figure 3. Picture of C7 ink (A) is rough and uneven while C16 ink (B) is smooth


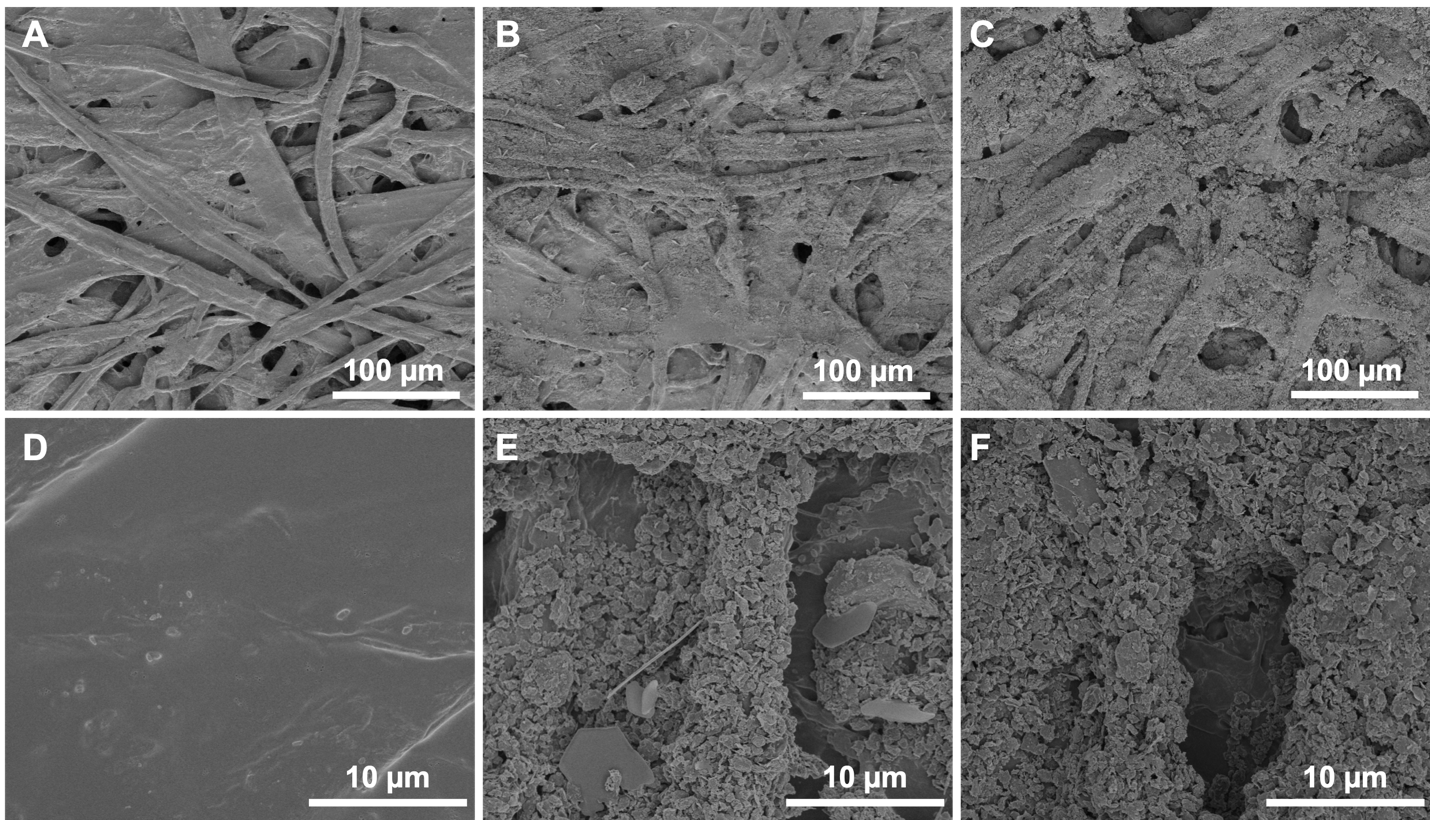


Figure 4. SEM showing graphene coating on the paper. Paper with 0 print (A and D); 1 print (B and E); 8 prints (C and F) of C16 ink top view.


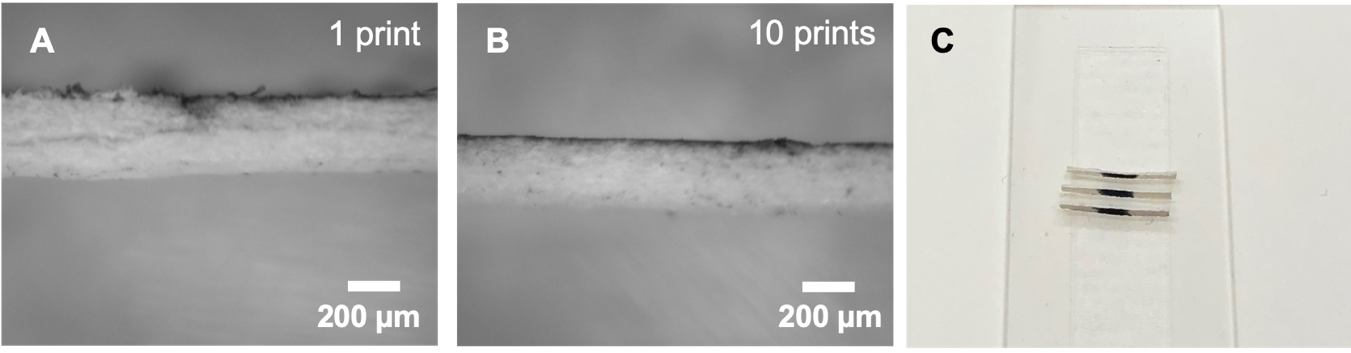


Figure 5. Optical microscope images of cross-section of paper with (A). 1 print, and (B).10 prints of C16 ink; (C) An image of the sample prepared for the microscope image showing the cross section facing the microscope.

The C16 ink was printed on paper 1 time and 10 time to compare the penetration depth of the ink on paper. The penetration depth of the ink on paper is studied by cutting a cross section of the paper shown in Figure 4C, and optical images of 1 print and 10 prints are shown in Figure 4A and B, and they exhibit about the same penetration depth.


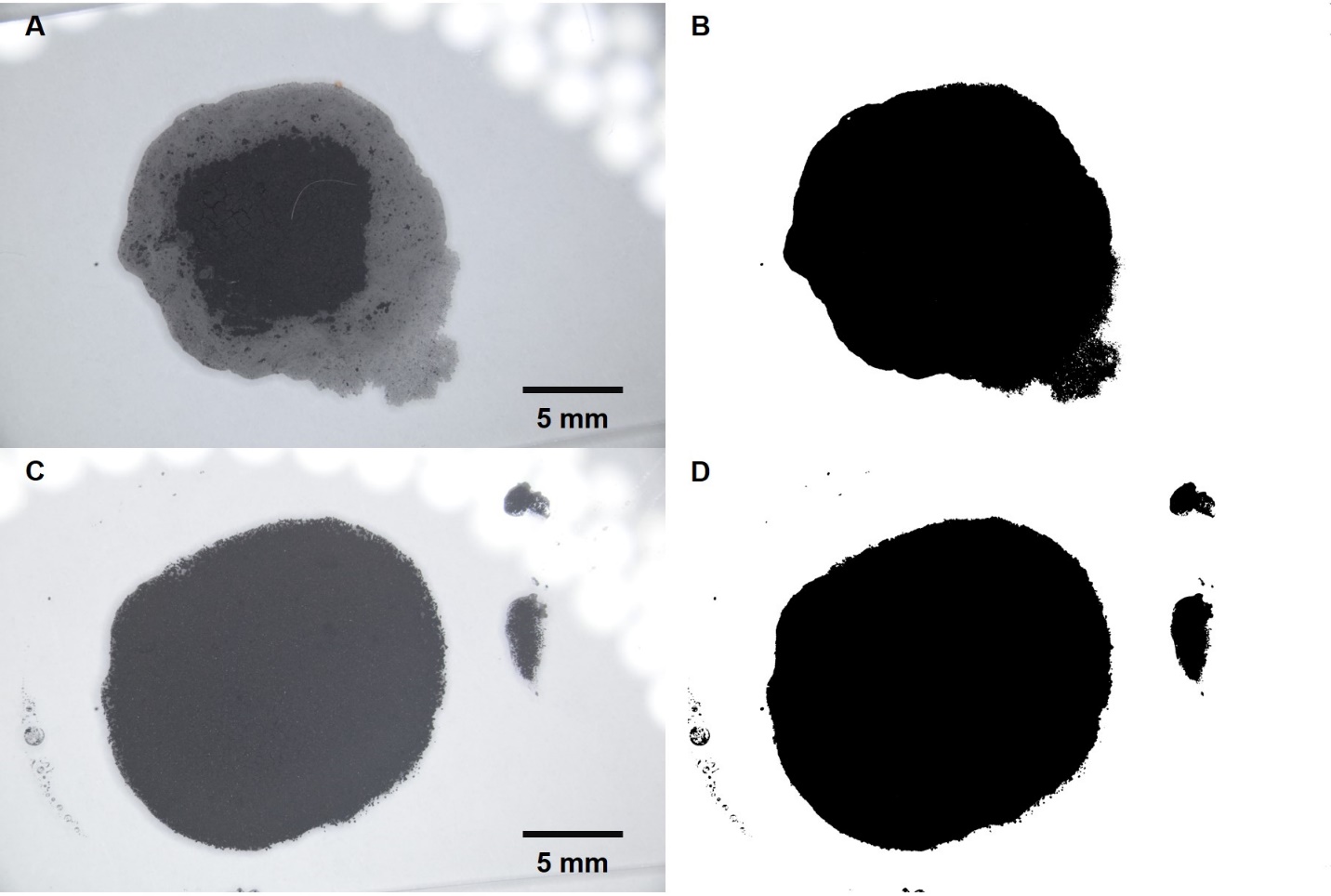


Figure 6. A dried C7 ink dot (A) and the area used for ink dot area calculation (B); A dried C16 ink dot (C) and the area used for ink dot area calculation (D).

C7 ink and C16 ink were drop cast on a glass slide and dried in a 90 C oven for a week. Sheet resistance of the ink were measured using the four-point probe method. The C7 ink dot is not uniform (Figure A), and appears to have two different thicknesses of the ink. The thinner graphene layer on the outside has sheet resistance from 40 kΩ/sq to 93 kΩ/sq, while the thicker graphene layer on the center has sheet resistance around 0.3 kΩ/sq. Area of the C7 ink dot was measured using ImageJ and is 2.06 cm^2^_._ Mass of the C7 ink dot was measured to be 1.1 mg, and since the density of the graphene is 2.266 g/cm^3^, the average thickness of the C7 ink dot is 2.36 μm. Hence, the average conductivity of the C7 ink is 10 S/m. Using the same concept, the average sheet resistance of C16 ink dot is 604 Ω/sq, and the sheet resistance is very uniform throughout the sample. The thickness of the ink dot is 5.8 μm, and the electrical conductivity of the C16 ink is 282 S/m.


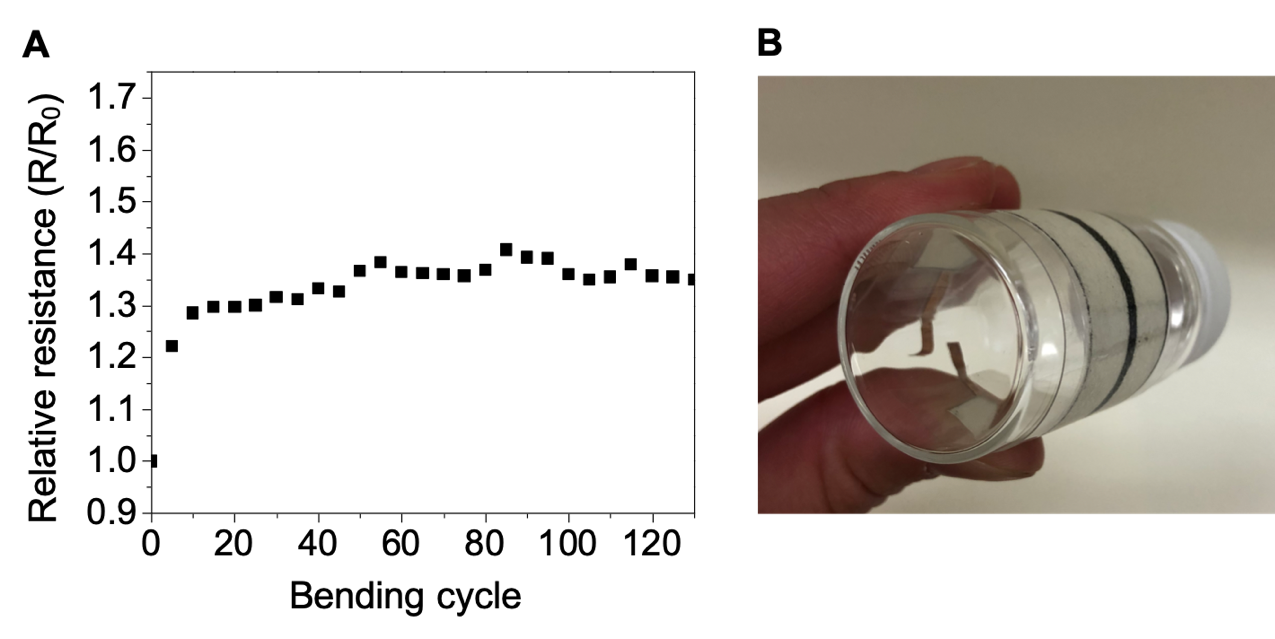


Figure 7. (A) Resistance change after the printed ink line was repeatedly rolled into a 7.62 mm diameter tube, in which R is the resistance after bending, and R_0_ is the original resistance of the ink line; (B) Illustration of bending the ink line.

C16 ink were printed and heated in the microwave with 10 s interval. Resistance of the ink line is measured using two-point probe method and results are shown in Figure 9. After 40 s microwaving, the resistance of the C16 ink line on paper are reduced into around 30% of its original resistance.


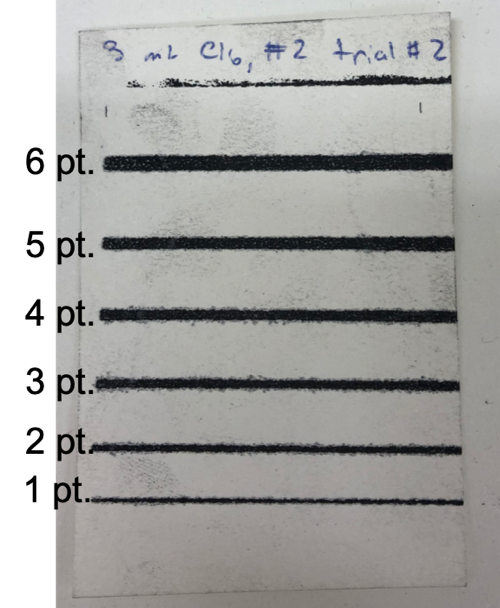


Figure 8. Picture of C16 ink lines (from 1 pt. to 6 pt.) with two prints.


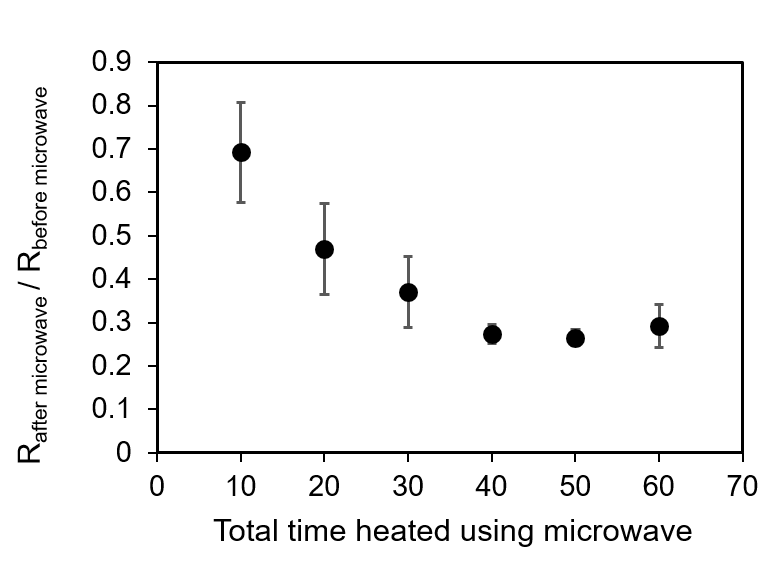


Figure 9. Resistance change after heating in the microwave for different amount of time


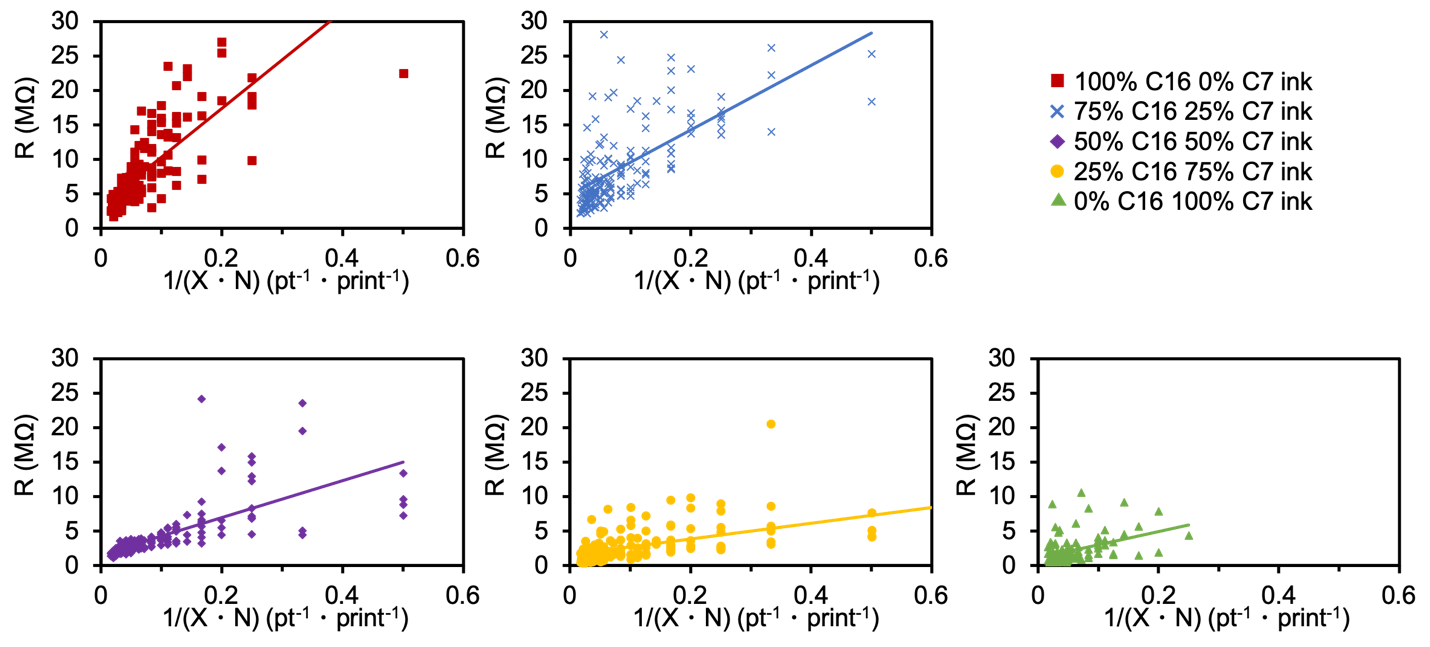

Figure 10. Full data sets of the electrical conductivity study.


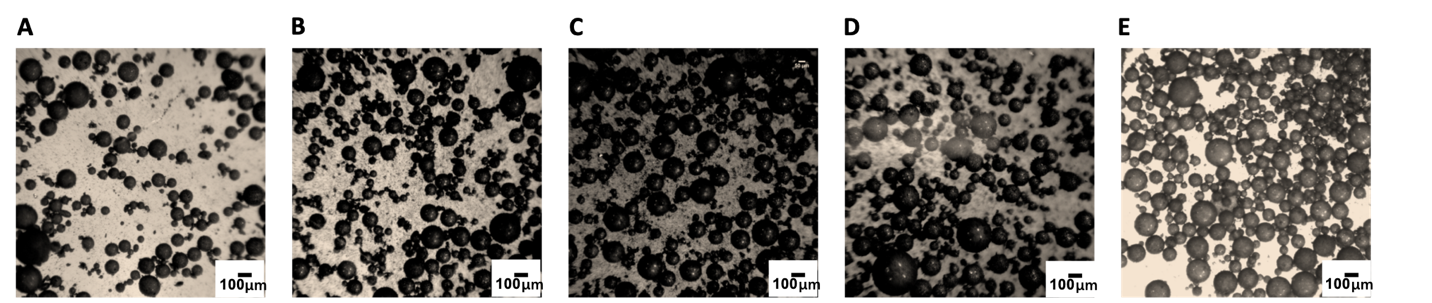


Figure 11. Optical microscope images of graphene ink prepared by using different ratio of C7/(C7+C16) volume% (each ink is diluted with the corresponding oil phase): (A) 100%; (B) 75%; (C) 50%; (D) 25%; (E) 0%.


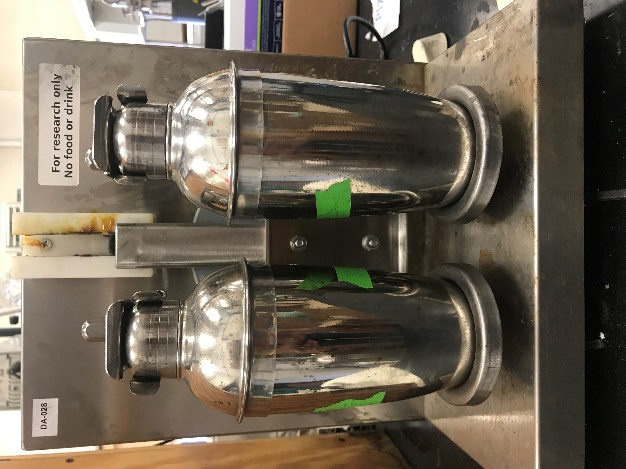


Figure 12. An image of the bubble tea shaker

Table 1 ǀ Resistance constant (*A*) of different inks and the uniformity value (R^2^) of different inks

| Ink | *A* (kΩ•print) | R^2^ |
| --- | --- | --- |
| 100% C16 and 0% C7 ink | 631.9 | 0.87 |
| 75% C16 and 25% C7 ink | 325.9 | 0.81 |
| 50% C16 and 50% C7 ink | 130.1 | 0.64 |
| 25% C16 and 75% C7 ink | 62.9 | 0.58 |
| 0% C16 and 100% C7 ink | 114.8 | 0.51 |

Table 2. Equations of the fitting lines of different inks.

| Ink | Fitting equation |
| --- | --- |
| 100% C16, 0% C7 ink | Y = 0.6319X + 0.0131 |
| 75% C16, 25% C7 ink | Y = 0.3259X + 0.0383 |
| 50% C16, 50% C7 ink | Y = 0.1301X + 0.0135 |
| 25% C16, 75% C7 ink | Y = 0.0629X + 0.0093 |
| 0% C16, 100% C7 ink | Y = 0.1148X + 0.0064 |
